# Vascular Changes and Surgical Risk in Cervical vs. Endometrial Cancer After Radiotherapy: A Retrospective Cohort Study

**DOI:** 10.3390/life16010071

**Published:** 2026-01-02

**Authors:** Daniela Marinescu, Laurențiu Augustus Barbu, Tiberiu Stefăniță Țenea Cojan, Nicolae-Dragoș Mărgăritescu, Liviu Vasile, Răzvan Alexandru Marinescu, Dumitru Sandu Ramboiu, Valeriu Șurlin, Ana-Maria Ciurea

**Affiliations:** 1 Department of Surgery, Emergency County Hospital, University of Medicine and Pharmacy of Craiova, 2 Petru Rares Street, 200349 Craiova, Romania; dtmarinescu@yahoo.com (D.M.); dmargaritescu@yahoo.com (N.-D.M.); vliviu777@yahoo.com (L.V.); sandu_r@yahoo.com (D.S.R.); 2 Department of Surgery, Railway Clinical Hospital Craiova, University of Medicine and Pharmacy of Craiova, 2 Petru Rares Street, 200349 Craiova, Romania; tiberiu.tenea@umfcv.ro; 3 Department of Plastic Surgery, University of Medicine and Pharmacy of Craiova, 200349 Craiova, Romania; razvanalexandrumarinescu98@gmail.com; 4 Department of Oncology, Emergency County Hospital, University of Medicine and Pharmacy of Craiova, 2 Petru Rares Street, 200349 Craiova, Romania; amciurea14@gmail.com

**Keywords:** radiotherapy, pelvic cancer, cervical cancer, endometrial cancer, perivascular fibrosis, inflammatory thrombosis, surgical risk, perioperative complications

## Abstract

Background: Radiotherapy is a cornerstone of treatment for cervical and endometrial cancers but is associated with vascular and perivascular changes that can increase surgical complexity and perioperative morbidity. While these effects are well documented in head, neck, and mediastinal irradiation, the pelvic vasculature remains underexplored. Methods: We retrospectively analyzed 119 patients who underwent pelvic oncologic surgery after RT (57.1% cervical cancer, 42.9% endometrial cancer). Intraoperative vascular findings were recorded and correlated with tumor type, perioperative complications, and vascular injury. Logistic regression was used to identify predictors of perioperative morbidity. Results: Perivascular fibrosis (21.8%) and inflammatory thrombosis (10.1%) were the most frequent intraoperative vascular changes, with no significant differences between tumor types. Most patients required no vascular procedure; when needed, simple venorrhaphy was sufficient, and no complex vascular reconstructions were performed. Perioperative complications occurred more frequently in cervical cancer patients (RR = 2.66; *p* = 0.02), with hemorrhage and urinary tract injury being the most common. Cervical tumor site and perivascular fibrosis were borderline predictors of complications. Conclusions: Neoadjuvant RT induces measurable intraoperative vascular changes without significantly increasing major vascular injury, particularly in experienced surgical settings. Cervical cancer patients represent a higher-risk subgroup, underscoring the need for meticulous surgical planning and multidisciplinary perioperative management. Perivascular fibrosis may serve as a marker for operative risk stratification, and long-term vascular surveillance is warranted due to the potential for delayed macrovascular events.

## 1. Introduction

Radiotherapy (RT) is a cornerstone of curative and adjuvant treatment in many pelvic and gynecologic malignancies. Although modern techniques have improved locoregional control and long-term survival, the vascular sequelae of RT are increasingly recognized as a significant contributor to morbidity in cancer survivors [1]. Radiation-induced vasculopathy encompasses a spectrum of vascular lesions affecting small, medium, and large vessels, characterized by endothelial injury, inflammatory activation, microthrombosis, and progressive perivascular fibrosis, ultimately leading to vessel wall fragility, stenosis, or aneurysm formation [2].

The underlying mechanisms involve persistent oxidative stress and NF-κB–mediated endothelial activation, promoting chronic inflammation and structural vascular remodeling [3]. Clinically, these changes can manifest early, through increased tissue fragility and bleeding during surgery, or late, as occlusive or aneurysmal disease developing years after treatment. Most evidence originates from head, neck, and mediastinal irradiation, where characteristic vasculopathic patterns and their clinical implications have been well documented. By contrast, the pelvic vasculature remains underexplored, despite the extensive use of RT in cervical and endometrial cancers and the recognized technical challenges associated with operating in previously irradiated fields [4].

In standard clinical practice, primary surgery followed by adjuvant radiotherapy ± chemotherapy is the preferred treatment sequence for most early-stage cervical and endometrial cancers. Surgery performed after radiotherapy is usually reserved for selected clinical scenarios, including locally advanced disease treated with neoadjuvant radiotherapy, residual or persistent disease, or radiation-related complications requiring surgical management. In these settings, surgical procedures are performed in previously irradiated fields and are therefore associated with increased technical complexity and potential perioperative risk.

In our cohort, most cervical cancer patients had locally advanced disease and were treated according to a neoadjuvant radiotherapy–first approach, followed by surgery with curative intent. Although most endometrial cancer patients were diagnosed at early stages, radiotherapy preceded surgery in selected cases based on individual risk assessment, tumor characteristics, or institutional multidisciplinary decision-making.

Pelvic oncologic surgery in this context is technically demanding due to fibrosis, distorted anatomical planes, and increased vascular fragility, which may elevate perioperative morbidity even in the absence of overt macrovascular disease [5]. Despite advances in oncologic management, surgical treatment of cervical and endometrial cancer in young women often entails radical procedures that inevitably lead to infertility, with a major impact on quality of life and long-term reproductive potential [6].

The aim of this study was to systematically characterize intraoperative vascular and perivascular changes in patients undergoing pelvic oncologic surgery after RT, to compare their frequency and impact between cervical and endometrial cancers, and to assess their association with perioperative complications. By integrating intraoperative findings with established pathophysiological mechanisms of radiation-induced vasculopathy, this study seeks to provide evidence to inform risk stratification, surgical planning, and multidisciplinary perioperative management.

## 2. Materials and Methods

### 2.1. Study Design and Setting

This retrospective observational cohort study was conducted at the First Clinic of Surgery, Clinical Emergency Hospital of Craiova, Romania, between January 2017 and December 2022. No patients were treated within a prospective clinical trial. All patients underwent pelvic oncologic surgery following preoperative pelvic radiotherapy (external beam ± vaginal brachytherapy), administered either as part of a neoadjuvant treatment strategy or for definitive management in selected clinical situations. The surgical procedures were performed by experienced gynecologic oncology teams, with vascular surgery support available when needed.

### 2.2. Patient Population

Surgery was performed after radiotherapy in all included patients, either as part of a neoadjuvant treatment strategy or for definitive management in selected clinical situations. A total of 119 patients were included: 68 with cervical cancer and 51 with endometrial cancer.

Cervical cancer group: mean age 52 years (range 29–78), 91% squamous carcinoma. Disease stage was assessed according to the International Federation of Gynecology and Obstetrics (FIGO) staging system, with stage distribution as follows: IB (32.35%), II (54.41%), and III (11.76%).

Endometrial cancer group: mean age 66 years (range 46–90), 91% endometrioid adenocarcinoma. Disease stage was assessed according to the FIGO staging system, with stage distribution as follows: I (62.75%), II (23.53%), and III (9.8%).

### 2.3. Inclusion and Exclusion Criteria

Eligible patients were those with histologically confirmed cervical or endometrial cancer who received preoperative pelvic radiotherapy (external beam ± vaginal brachytherapy), administered either as part of a neoadjuvant treatment strategy or for definitive management in selected clinical situations, and subsequently underwent pelvic oncologic surgery with curative intent at the First Clinic of Surgery, Clinical Emergency Hospital of Craiova, between January 2017 and December 2022. All included patients had complete perioperative and intraoperative data available for analysis. Patients were excluded if they presented with recurrent or metastatic disease at the time of surgery, had a history of pelvic exenteration or major vascular reconstruction, lacked complete medical or operative records, had previous non-oncologic pelvic radiation, or did not have pathological confirmation of malignancy.

### 2.4. Radiotherapy Protocol

All patients received external pelvic radiotherapy (40–45 Gy). Vaginal brachytherapy was administered selectively in a limited number of cases, primarily in cervical cancer patients and in selected high-risk endometrial cancer cases, based on multidisciplinary oncologic decision-making. Radiotherapy was administered preoperatively for downstaging in advanced disease, and surgical intervention was performed 6–8 weeks after completion of radiotherapy.

### 2.5. Surgical Procedures

All patients underwent total colpohysterectomy with bilateral adnexectomy.

In the cervical cancer group, 95.6% underwent pelvic lymphadenectomy and 10.3% paraaortic lymphadenectomy.

In the endometrial cancer group, 66.7% underwent pelvic lymphadenectomy, and paraaortic dissection was performed selectively in patients with suspected nodal invasion.

Vascular injuries were managed intraoperatively by simple venorrhaphy or primary repair in cases with <30% narrowing of the vena cava.

### 2.6. Intraoperative Vascular Assessment

Intraoperative assessment of post-radiation vascular changes was performed by direct surgical inspection and palpation during pelvic dissection and lymphadenectomy. No routine intraoperative venography, fluoroscopic imaging, or Doppler ultrasound was used. Radiation-related vascular alterations were identified based on macroscopic findings, including perivascular fibrosis, inflammatory reactions of the vascular sheath, venous wall fragility, and inflammatory thrombus formation, as documented during surgery.

Intraoperative findings focused on radiation-induced vascular changes, including

- fibrotic thickening of the iliac vascular sheath,
- inflammatory thrombus in iliac or cava veins,
- fragile venous wall injuries during lymphadenectomy.

No complex vascular reconstructions were required.

### 2.7. Surgical Perioperative Complications

Complications were recorded prospectively and categorized as intraoperative (e.g., vascular injury, hemorrhage, ureteral/bladder trauma) or postoperative (e.g., fistula, venous thrombosis).

### 2.8. Statistical Analysis

The statistical analysis aimed to compare clinical and intraoperative findings between groups and to identify factors associated with perioperative complications. Descriptive statistics were used to summarize baseline demographic, clinical, and intraoperative characteristics. Factors analyzed as potential predictors of surgical risk included tumor site (cervical vs. endometrial), patient age, perivascular fibrosis, inflammatory thrombosis, and intraoperative vascular injury. Continuous variables were expressed as means ± standard deviation (SD) or medians and interquartile ranges (IQR) depending on distribution, and categorical variables as absolute frequencies and percentages.

Group comparisons between cervical and endometrial cancer patients were performed using the chi-square test or Fisher’s exact test for categorical variables and the Mann–Whitney U test for continuous variables. To assess the association between vascular findings and perioperative complications, relative risks (RR) and odds ratios (OR) were calculated with 95% confidence intervals (CI). Binary logistic regression analysis was used to identify independent predictors of perioperative complications, including tumor site, age, perivascular fibrosis, inflammatory thrombosis, and intraoperative vascular injury. All statistical tests were two-tailed, and a *p*-value < 0.05 was considered statistically significant. Statistical analyses were performed using SPSS version 27.0 (IBM Corp., Armonk, NY, USA). Data preprocessing and descriptive analyses were conducted using Microsoft Excel 2016 (Microsoft Corp., Redmond, WA, USA) with XLSTAT 2022 (Addinsoft SARL, Paris, France).

### 2.9. Ethical Approval

The study was conducted according to the Declaration of Helsinki and approved by the Ethics Committee of the Clinical Emergency Hospital of Craiova (approval no. 55513/ 18 November 2025). Informed consent was waived due to the retrospective nature of the study, and data were anonymized.

## 3. Results

Cervical cancer was more common than endometrial cancer (57.1% vs. 42.9%). Squamous carcinoma was the predominant histological type, followed by endometrioid adenocarcinoma (Table 1).

Most patients were aged 50 years or older, with the highest proportion in the 50–59 age group (Table 2).

HPV 16 was the most prevalent genotype, followed by negative cases and HPV 18, indicating a clear predominance of HPV 16 among patients (Table 3).

**Figure 1:**
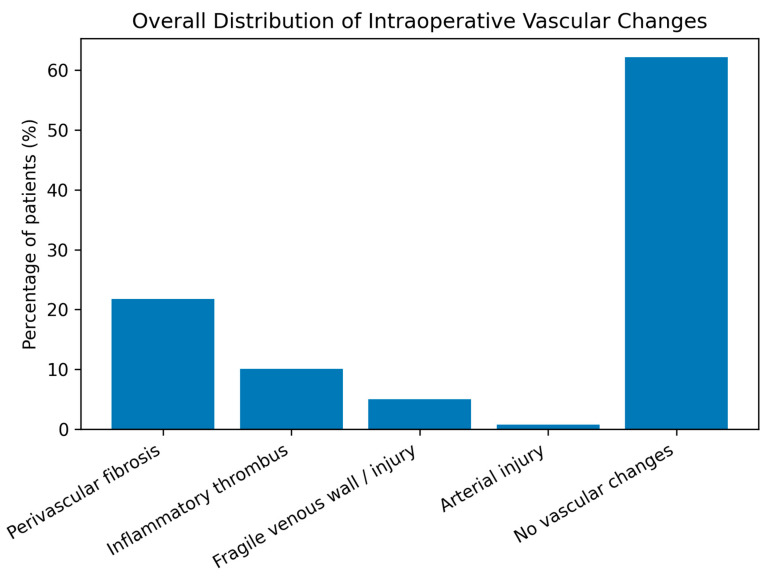
Overall distribution of intraoperative radiation-induced vascular changes in the study cohort.

**Figure 2:**
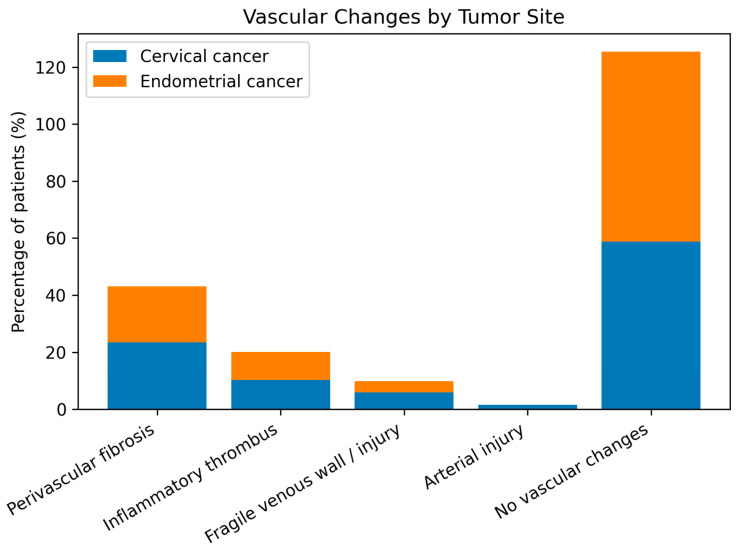
Comparison of intraoperative vascular changes between cervical and endometrial cancer patients after radiotherapy.

Radiation-induced vascular changes were common, with perivascular fibrosis and inflammatory thrombus being the most frequent findings, particularly in cervical cancer patients. Most cases showed no significant vascular alterations (Table 4, Figure 1 and Figure 2).

**Figure 3:**
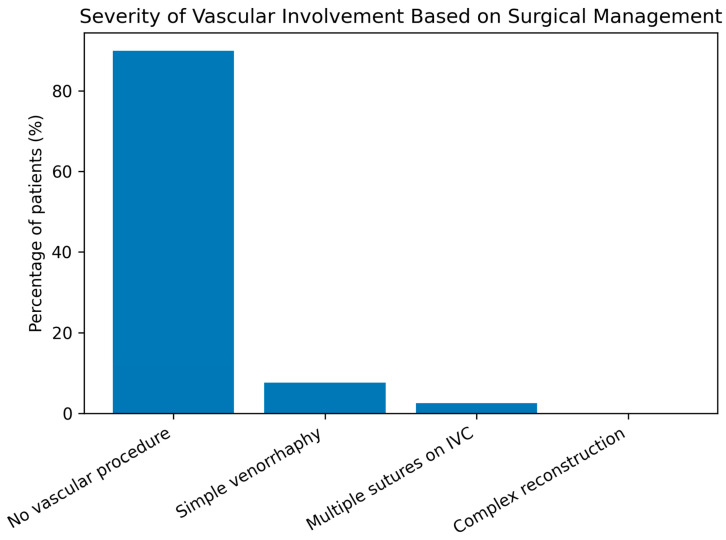
Severity of radiation-induced vascular involvement assessed by the need for intraoperative vascular procedures.

Most cases required no vascular procedure, and when needed, simple venorrhaphy was the most common intervention. No complex vascular reconstructions were performed (Table 5, Figure 3).

Perioperative complications were more frequent in cervical cancer patients, with bleeding and urinary tract injuries being the most common, while most patients had no complications (Table 6).

As expected, the cohort included a higher proportion of cervical cancer patients compared to endometrial cancer patients (57.1% vs. 42.9%). As expected, histological type differed significantly between cervical and endometrial cancer patients, reflecting the well-known biological characteristics of these tumor entities, with squamous carcinoma predominating in cervical cancer and endometrioid adenocarcinoma in endometrial cancer (Table 7). In addition, HPV status and age distribution differed significantly between groups, with HPV 16 predominating in cervical cancer patients and endometrial cancer patients being significantly older.

Cervical cancer patients had a significantly higher relative risk of perioperative complications (RR = 2.66), while the relative risk of vascular injury (RR = 1.65) was not statistically significant (Table 8).

To identify factors associated with increased surgical risk, univariate and multivariate analyses were performed (Table 8, Table 9 and Table 10). Cervical tumor site and perivascular fibrosis emerged as borderline predictors of perioperative complications.

The relative risk of perioperative complications was significantly higher in cervical cancer patients (RR = 2.66), while the relative risk of vascular injury (RR = 1.65) showed no significant difference between groups (Table 9).

In multivariate logistic regression (Table 10), cervical tumor site and perivascular fibrosis showed borderline associations with perioperative complications (OR 2.58, 95% CI 0.89–7.48, *p* = 0.082; OR 2.37, 95% CI 0.88–6.40, *p* = 0.088). Age, inflammatory thrombus, and vascular injury were not significant predictors.

## 4. Discussion

### 4.1. Comparison with Literature

Radiotherapy is well known to induce both structural and functional vascular alterations through endothelial injury, inflammatory activation, and progressive fibrotic remodeling. The observed difference in histological type between tumor sites reflects established tumor biology rather than a novel finding, and was therefore not further explored as an independent predictor of perioperative outcomes. Several studies have shown that ionizing radiation triggers endothelial dysfunction, microthrombosis, and perivascular fibrosis, ultimately accelerating atherosclerosis and contributing to vessel wall fragility over time [7,8]. This mechanism aligns with the histopathological findings in our pelvic cohort, where perivascular fibrosis and inflammatory thrombus were frequent, despite no significant increase in major vascular injuries.

Similar vascular changes have been described across other anatomical regions, particularly the head, neck, and mediastinum. Both historical and contemporary reports document periarterial fibrosis and chronic thrombosis, occasionally associated with delayed stenoses or strictures, even in the absence of recurrent disease [2,9]. This pattern supports the concept of a fibrotic vascular signature characteristic of irradiated tissues. Among the factors analyzed, cervical tumor site and perivascular fibrosis were associated with a higher surgical risk, supporting the role of radiation-induced tissue changes rather than overt vascular injury.

Surgically, irradiated fields are recognized as technically challenging because of dense fibrosis, loss of dissection planes, and increased friability However, the literature indicates that complex vascular reconstructions are rarely required in irradiated fields, as most intraoperative vascular issues can be managed conservatively or with simple surgical repair, such as venorrhaphy [9]. When used, intraoperative venography serves as a diagnostic tool to guide immediate decision-making rather than as a corrective intervention. Our results parallel these findings, with a low rate of vascular reconstruction despite frequent perivascular changes.

Cardio-oncology studies further highlight that perioperative morbidity is increased in irradiated patients, driven largely by tissue fragility and hemorrhagic risk rather than by overt vascular injury [8,10]. This is consistent with our finding of a higher complication rate in cervical cancer patients, likely reflecting altered tissue quality rather than macrovascular events.

Large vessel complications after RT, such as stenosis, occlusion, or aneurysm, are well documented but tend to occur later and are strongly influenced by dose, field, and latency [11]. Ophthalmic and neurovascular series show that radiation-associated occlusive events may arise years or decades after treatment, often accompanied by extensive collateral formation [12,13,14]. Pediatric studies, including proton radiotherapy cohorts, report moyamoya-like vasculopathy in 6–7% of patients after several years, emphasizing that macrovascular events are selective and delayed, whereas early changes are predominantly microvascular and fibrotic [15].

From an ophthalmologic perspective, post-radiation retinopathy further illustrates this chronology. Occlusive capillaropathy and ischemia typically develop after long latency, especially at higher doses, while choroidal histology reveals hyalinization, ectasia, and pathologic neovascularization [16,17]. Similar mechanisms likely underlie the fibrotic, friable vascular tissue encountered intraoperatively in the pelvis.

Experimental and translational studies reinforce this model. NF-κB–driven inflammatory signaling, oxidative stress, and intimal hyperplasia have been implicated in radiation-induced vascular injury [18,19]. Genetic susceptibility may also modulate risk, although no single determinant has been identified [20].

Management strategies reported in neurovascular literature emphasize a tailored, minimally invasive approach. Endovascular techniques are often preferred over open repair because of poor tissue planes and fragility [2,21]. This is fully consistent with our experience, where no major vascular reconstructions were required. Additionally, adjuvant factors such as cisplatin therapy may potentiate vascular inflammation, as illustrated in leukocytoclastic vasculitis case reports [22].

Our pelvic cohort mirrors the well-characterized vascular signature of post-radiation injury—endothelial dysfunction, perivascular fibrosis, and inflammatory thrombosis—described across neurovascular, head-and-neck, and mediastinal series. Despite frequent local vascular changes, major intraoperative vascular reconstructions were rarely required. The higher perioperative complication rate in cervical cancer patients likely reflects tissue quality changes rather than overt macrovascular injury. These findings align with literature showing that early radiation effects are predominantly microvascular and fibrotic, while major occlusive events are selective and delayed, and with evidence implicating NF-κB–driven chronic inflammation and dose-dependent tissue fragility.

### 4.2. Mechanisms of Complications

Radiotherapy induces a complex cascade of vascular injury involving endothelial dysfunction, chronic inflammation, microthrombosis, and progressive fibrotic remodeling, which together increase perioperative morbidity even in the absence of overt macrovascular lesions [23].

Ionizing radiation causes direct damage to endothelial cells through the generation of reactive oxygen species (ROS) and activation of inflammatory pathways such as NF-κB, leading to increased vascular permeability, exposure of subendothelial structures, and activation of the coagulation cascade. This endothelial dysfunction sets the stage for local thrombosis and chronic vascular remodeling [18,19].

Radiation injury is not limited to an acute effect but triggers a chronic inflammatory response sustained by macrophages, lymphocytes, and profibrotic mediators such as TGF-β and VEGF. This leads to intimal thickening, perivascular collagen deposition, and fibrotic transformation of the vessel wall. Intraoperatively, these processes manifest as fibrotic, adherent, and poorly dissectible vessels [14,24].

Endothelial injury promotes the expression of adhesion molecules, platelet activation, and fibrin deposition, resulting in inflammatory microthrombi in small and medium vessels. These changes can increase perioperative bleeding risk, local tissue ischemia, and impaired wound healing even when no major vascular lesion is present [8,12,25].

Chronic remodeling ultimately leads to perivascular fibrosis, loss of normal anatomical planes, and increased vessel wall fragility. These features make dissection technically challenging and increase the risk of intraoperative vascular injury without a proportional rise in major vascular reconstruction rates [2,9,26].

Over time, these mechanisms can lead to progressive stenosis, occlusion, or aneurysm formation in irradiated vessels. Such events typically occur years to decades after treatment and are strongly influenced by radiation dose, field, and latency [15,21].

Systemic factors further modulate these vascular changes. Cisplatin-based chemoradiotherapy has been associated with leukocytoclastic vasculitis, reflecting an exacerbation of vascular inflammation [22,27]. Classical cardiovascular risk factors (e.g., hypertension, dyslipidemia, smoking) and genetic susceptibility may also increase vulnerability to radiation vasculopathy [20,28].

Perioperative morbidity is increased in irradiated patients because fibrosis makes vessels fragile and adherent, microthrombotic changes impair hemostasis and wound healing, and major vascular complications typically occur in a delayed rather than immediate perioperative phase [29].

This pathophysiological framework is consistent with our finding of increased perioperative complications but low rates of vascular reconstruction, and underscores the need for tailored surgical planning and multidisciplinary perioperative management.

### 4.3. Clinical Implications

The vascular and perivascular changes induced by radiotherapy have significant surgical and perioperative consequences. Even in the absence of major vascular stenosis or occlusion, irradiated tissues demonstrate increased fragility, altered anatomical planes, and a prothrombotic and proinflammatory environment, all of which can influence the safety and complexity of pelvic oncologic surgery.

Although individual patient characteristics such as comorbidities, functional status, and lifestyle factors are important determinants of surgical risk, these data were not uniformly available in this retrospective cohort. In this context, tumor site and intraoperative markers of radiation-induced tissue damage, such as perivascular fibrosis, represent practical and readily identifiable indicators of increased surgical risk.

Fibrotic remodeling and loss of normal tissue planes make dissections technically challenging, leading to increased intraoperative bleeding, a higher risk of inadvertent vascular injury, and more difficult hemostasis in irradiated fields [30]. Although major vascular reconstructions are rarely needed, surgical teams must anticipate challenging dissections, fragile vessels, and atypical tissue consistency [2,9,31]. This principle is consistent with evidence from adnexal surgery, where accurate intraoperative assessment and recognition of benign adnexal lesions, such as ovarian serous papillary cysts, have been shown to reduce unnecessary radical interventions and perioperative morbidity, while preserving reproductive potential in appropriate patients [32].

Multiple studies show that irradiated patients have higher rates of perioperative complications, largely related to tissue friability, impaired wound healing, and hemorrhagic risk rather than overt macrovascular events. This is consistent with our finding of a higher complication rate in cervical cancer patients, despite similar rates of major vascular injury compared to controls [8,10,33,34].

These findings underscore the critical role of meticulous surgical technique, early recognition of vulnerable vessels, and access to experienced vascular support during pelvic oncologic surgery.

Macrovascular events (e.g., stenosis, occlusion, pseudoaneurysm) typically occur years after radiation, highlighting the importance of long-term vascular surveillance even in asymptomatic patients, maintaining a high index of suspicion during follow-up, and integrating vascular imaging into survivorship care when appropriate [15,35].

When vascular complications do occur, the literature supports a preference for endovascular management over open repair in irradiated fields, due to the fragility of the tissues and the higher risk of surgical complications [15,36]. This principle can be extrapolated to the pelvis: conservative or endovascular interventions should be considered first-line whenever feasible.

Given the interplay between oncologic treatment, vascular changes, and surgical complexity, optimal patient care necessitates preoperative evaluation with vascular and anesthesia teams, individualized surgical planning based on prior radiation exposure, proactive perioperative hemostatic strategies, and immediate access to interventional radiology for rapid response.

Furthermore, adjuvant systemic therapies such as cisplatin may amplify vascular fragility [22] cisplatin, supporting the need for integrated oncologic–vascular risk stratification. Individual variability—including baseline cardiovascular comorbidities and possible genetic susceptibility—can modulate the severity of radiation vasculopathy [20,37]. Incorporating these factors into clinical decision-making enables the identification of high-risk patients before surgery, the implementation of tailored intraoperative precautions, and the establishment of structured vascular follow-up for early detection of delayed complications.

Irradiated pelvic fields necessitate anticipation of surgical challenges, even in the absence of macrovascular stenosis, as complications often stem from compromised tissue quality rather than overt vascular occlusion. When intervention is required, endovascular strategies should be prioritized, and long-term vascular monitoring is recommended due to the risk of delayed events. Multidisciplinary perioperative planning enhances patient safety, while individualized risk profiling can help optimize surgical and long-term outcomes.

In this cohort of patients with pelvic malignancies treated with radiotherapy, several clinically relevant patterns were observed. Cervical cancer predominated over endometrial cancer (57.1% vs. 42.9%), reflecting the known epidemiologic and virologic profile of the disease, while endometrial cancer patients were significantly older, highlighting distinct risk factors and tumor biology. Intraoperative vascular alterations such as perivascular fibrosis (≈22%) and inflammatory thrombus (≈10%) were common but did not differ significantly between tumor types. Although complex vascular reconstructions were rarely required, the frequent need for conservative management or simple venorrhaphy underscores the technical impact of these changes. Perioperative complications were significantly more frequent in cervical cancer patients (RR = 2.66; *p* = 0.02), particularly hemorrhage and urinary tract injury, reflecting higher surgical morbidity. Tumor site (cervical) and perivascular fibrosis emerged as borderline predictors of complications (OR ≈ 2.5), suggesting an indirect effect of post-radiation tissue changes on operative risk, while age, inflammatory thrombosis, and macrovascular lesions were not independent predictors, indicating that perioperative morbidity is driven more by tissue quality than by overt vascular pathology.

### 4.4. Strengths and Limitations

This study has several important strengths. First, it focuses on a well-defined and clinically relevant cohort of patients undergoing pelvic oncologic surgery after radiotherapy, reflecting real-world surgical practice in a tertiary cancer center. Second, the integration of clinical data and detailed intraoperative assessment provides a comprehensive characterization of radiation-induced vascular and perivascular changes, helping to bridge the gap between known pathophysiological mechanisms and surgical outcomes. Third, the direct comparison between cervical and endometrial cancer patients offers valuable insights into tumor-specific perioperative risk profiles, which have been insufficiently addressed in previous literature. Finally, the standardized surgical approach and detailed intraoperative vascular documentation enhance the internal validity and reproducibility of the findings.

However, several limitations should be acknowledged. The retrospective and single-center design may introduce selection bias and limit the generalizability of the results. The absence of detailed data on comorbidities, lifestyle factors, and functional status limited a more granular patient-level risk stratification. The sample size, while representative for a specialized surgical cohort, may not provide adequate statistical power to detect subtle differences in rare vascular events. In addition, the perioperative observation window was short, precluding conclusions regarding long-term vascular sequelae, which are known to manifest years after radiotherapy. Vascular alterations were assessed primarily intraoperatively, without systematic pre- and postoperative vascular imaging, which may have underestimated subclinical or progressive changes. Moreover, the absence of detailed dosimetric and dose–volume data limits the ability to explore potential dose–response relationships.

Despite these limitations, the present study provides clinically meaningful and original evidence on the vascular impact of pelvic radiotherapy. Its findings support more refined surgical planning, risk stratification, and multidisciplinary perioperative management in patients with gynecologic malignancies previously treated with radiotherapy.

## 5. Conclusions

This study demonstrates that neoadjuvant radiotherapy induces measurable intraoperative vascular changes—most commonly perivascular fibrosis and inflammatory thrombosis—without significantly increasing the risk of major vascular injury, particularly when managed by experienced surgical teams. Cervical cancer patients, however, exhibited a higher perioperative complication rate, highlighting their classification as a higher-risk subgroup and underscoring the value of careful preoperative planning and the availability of a multidisciplinary team, including vascular surgery support when needed.

Identification and documentation of perivascular fibrosis may serve as a useful marker for perioperative risk stratification, guiding intraoperative decision-making. These findings are consistent with the characteristic fibrotic and microthrombotic vascular signature described in radiation-induced vasculopathy and emphasize the importance of anticipating tissue fragility in irradiated fields.

Incorporating structured risk assessment, tailored surgical strategies, and long-term vascular surveillance may improve perioperative safety and outcomes in patients undergoing pelvic oncologic surgery after RT. Future studies integrating dosimetric parameters and molecular profiling could further refine risk prediction models.

## Figures and Tables

**Table 1:** Tumor site and histological type distribution.

Tumor Site/Histological Type	Number of Patients	% of Total
**Tumor site**		
**Endometrial**	51	42.9%
**Cervical**	68	57.1%
**Histological type**		
**Squamous carcinoma**	62	52.1%
**Endometrioid adenocarcinoma**	52	43.7%
**Serous/clear/mixed types**	5	4.2%

**Table 2:** Age group distribution.

Age Group (Years)	Number of Patients	%
**<40**	14	11.8
**40–49**	26	21.8
**50–59**	41	34.5
**≥60**	38	31.9

**Table 3:** HPV status.

HPV Type	Number of Patients	% of Total
**16**	64	47.4%
**18**	11	8.1%
**Negative**	60	44.5%

**Table 4:** Intraoperative vascular changes observed in patients with pelvic tumors after neoadjuvant radiotherapy.

Vascular Change	Cervical Cancer (n = 68)	Endometrial Cancer (n = 51)	Total (%)
**Perivascular fibrosis (iliac/cava)**	16 (23.5%)	10 (19.6%)	21.8%
**Inflammatory thrombus (EIV/CIV)**	7 (10.3%)	5 (9.8%)	10.1%
**Fragile venous wall/intraoperative injury**	4 (5.9%)	2 (3.9%)	5.0%
**Arterial injury**	1 (1.5%)	0	0.8%
**No vascular changes**	40 (58.8%)	34 (66.7%)	62.2%

**Table 5:** Types of intraoperative vascular procedures performed.

Type of Vascular Procedure	Cervical Cancer (n = 68)	Endometrial Cancer (n = 51)	Total (%)
**Simple venorrhaphy**	6 (8.8%)	3 (5.9%)	7.6%
**Multiple sutures on IVC**	2 (2.9%)	1 (2.0%)	2.5%
**Complex reconstruction (graft)**	0	0	0%
**No vascular procedure**	60 (88.2%)	47 (92.1%)	89.9%

**Table 6:** Perioperative complications.

Complication	Cervical Cancer (n = 68)	Endometrial Cancer (n = 51)	Total (%)
**Major intraoperative bleeding**	3 (4.4%)	1 (2.0%)	3.3%
**Ureter or bladder injury**	4 (5.8%)	2 (3.9%)	5.0%
**Postoperative venous thrombosis**	2 (2.9%)	1 (2.0%)	2.5%
**Urinary fistula**	2 (2.9%)	0	1.7%
**Other complications**	1 (1.5%)	0	0.8%
**No complications**	56 (82.3%)	47 (92.2%)	86.6%

**Table 7:** Statistical analysis of distribution and comparison between cervical and endometrial cancer groups.

Variable	Statistical Test	Test Value (χ^2^/U)	*p*-Value	Significance	Interpretation
**Tumor site**	Chi-square goodness of fit	χ^2^ = 47.26	5.46 × 10^−11^	***p* < 0.001**	There is a significant difference in the distribution of tumor site.
**Histological type**	Chi-square test of independence	χ^2^ = 276.00	2.33 × 10^−49^	***p* < 0.001**	Histological type distribution differs significantly between tumor sites.
**Age (years)**	Mann–Whitney U test	U = 1348.5	0.00038	***p* < 0.001**	Patients with endometrial cancer are significantly older.
**HPV status**	Chi-square test of independence	χ^2^ = 257.57	9.89 × 10^−53^	***p* < 0.001**	HPV distribution differs significantly between the two cancer groups.

**Table 8:** Statistical analysis of intraoperative vascular changes and perioperative complications in cervical versus endometrial cancer patients.

Variable	Statistical Test	*p*-Value	Significance	Interpretation
**Perivascular fibrosis**	Chi-square	0.490	n.s.	No significant difference between groups
**Inflammatory thrombus**	Chi-square	0.633	n.s.	No difference between groups
**Vascular injury**	Chi-square	0.656	n.s.	Similar distribution between groups
**Perioperative complications**	Chi-square	0.055	borderline	Slightly more frequent in cervical cancer patients
**Type of complications**	Chi-square	0.040	significant	Distribution of complication types differs between groups

Note: *p* < 0.05 was considered statistically significant. n.s. = not significant.

**Table 9:** Relative risk (RR) and odds ratio (OR) for intraoperative vascular injury and perioperative complications in cervical versus endometrial cancer patients.

Outcome	Cervical (Yes/Total)	Endometrial (Yes/Total)	RR (95% CI)	OR (95% CI)	*p*-Value	Interpretation
**Vascular injury**	7/67	4/63	1.65 (0.51–5.35)	1.72 (0.48–6.19)	n.s.	No significant difference in vascular injury risk between groups
**Perioperative complications**	17/67	6/63	2.66 (1.12–6.33)	3.23 (1.18–8.83)	0.02	Cervical cancer patients had significantly higher complication risk

Note: RR = relative risk; OR = odds ratio; CI = confidence interval; n.s. = not significant (*p* > 0.05). A two-tailed significance level of *p* < 0.05 was used.

**Table 10:** Binary logistic regression analysis of perioperative complication predictors.

Variable	OR (95% CI)	*p*-Value	Interpretation
**Tumor site (cervical)**	2.58 (0.89–7.48)	0.082	Borderline ↑ risk of complications
**Age (years)**	0.98 (0.93–1.02)	0.256	NS
**Perivascular fibrosis**	2.37 (0.88–6.40)	0.088	Borderline ↑ risk
**Inflammatory thrombus**	0.81 (0.15–4.28)	0.801	NS
**Vascular injury**	2.45 (0.59–10.20)	0.217	NS

Note: OR = odds ratio; CI = confidence interval; NS = not significant. *p* < 0.05 was considered statistically significant, ↑ indicates an increased risk of complications.

## Data Availability

The data presented in this study are available on request from the corresponding author. The data are not publicly available due to patient confidentiality.
